# Unraveling the Variability of Human Satiation: Implications for Precision Obesity Management

**DOI:** 10.21203/rs.3.rs-4402499/v1

**Published:** 2024-05-23

**Authors:** Andres Acosta, Lizeth Cifuentes, Diego Anazco, Timothy O’Connor, Maria Hurtado, Wissam Ghusn, Alejandro Campos, Sima Fansa, Alison McRae, Sunil Madhusudhan, Elle Kolkin, Michael Ryks, William Harmsen, Barham Abu Dayyeh, Donald Hensrud, Michael Camilleri

**Affiliations:** Mayo Clinic; Mayo Clinic; Mayo Clinic; Phenomix Sciences Inc; Mayo Clinic; Mayo Clinic; Mayo Clinic; Mayo Clinic; Mayo Clinic; Phenomix Sciences Inc; Phenomix Sciences Inc; Mayo Clinic; Mayo Clinic; Mayo Clinic; Mayo Clinic; Mayo Clinic College of Medicine

## Abstract

Satiation is the physiologic process that regulates meal size and termination, and it is quantified by the calories consumed to reach satiation. Given its role in energy intake, changes in satiation contribute to obesity’s pathogenesis. Our study employed a protocolized approach to study the components of food intake regulation including a standardized breakfast, a gastric emptying study, appetite sensation testing, and a satiation measurement by an *ad libitummeal* test. These studies revealed that satiation is highly variable among individuals, and while baseline characteristics, anthropometrics, body composition and hormones, contribute to this variability, these factors do not fully account for it. To address this gap, we explored the role of a germline polygenic risk score, which demonstrated a robust association with satiation. Furthermore, we developed a machine-learning-assisted gene risk score to predict satiation and leveraged this prediction to anticipate responses to anti-obesity medications. Our findings underscore the significance of satiation, its inherent variability, and the potential of a genetic risk score to forecast it, ultimately allowing us to predict responses to different anti-obesity interventions.

Obesity, a chronic and complex disease of epidemic proportions, affects over 650 million adults worldwide^1^. Obesity’s pathophysiology is heterogeneous and influenced by complex interactions between genetic, environmental, and behavioral factors. This heterogeneity has been reflected in the well documented variable response to all anti-obesity interventions among individuals with obesity^2^. While unraveling the intricacies of obesity remains a significant challenge, this understanding is critical for developing individualized and more effective treatment strategies. Recent advancements have led to the recognition of distinct obesity phenotypes based on the multifaceted pathophysiology of obesity^3^. Consequently, targeting specific underlying pathophysiologic processes has shown promising results in response to different weight loss interventions^3–6^.

At the core of obesity’s pathogenesis, lies the concept of energy imbalance, which results in excess energy accumulation in the form of increased adipose tissue. Physiologically, energy balance is a dynamic interplay between food intake and energy expenditure. The food intake cycle stages are hunger, satiation, and postprandial satiety^7^. Hunger is characterized by the pre-prandial desire to eat and it is described as an uncomfortable emptiness in the abdomen that leads to meal initiation^8^. Satiation is characterized by a prandial feeling of fullness that regulates the meal size and termination. Satiation is quantified by the calories consumed before reaching a usual level of fullness – or satiation^7,9,10^. Postprandial satiety is characterized by the postprandial sensation of fullness after meal termination, and determines the timing for the next meal^11^. Although these terms imply that food intake regulation is a step-by-step system, this is not necessarily the case as there is an inter-meal period in which neither fullness nor hunger are dominating. Nonetheless, any disruption in this cycle can lead to excess energy intake, increasing the risk of obesity.

Satiation has emerged as a promising avenue for precision obesity management, potentially paving the way to enhance the weight loss response to current antiobesity interventions. Hence it is important to understand the variability of satiation. Our team has developed a validated comprehensive deep-phenotyping protocol to measure hunger, satiation, and postprandial satiety^3,4^. In this 10-hour long, deep-phenotyping protocol, individuals are asked to eat until reaching maximal fullness or satiation, assessed by visual analog scales. The primary measure is the total number of calories consumed to reach satiation (CTS) that leads to meal termination^12^. This validated satiation measurement tool provides insights into satiation dynamics and its potential impact on obesity management. In humans, higher CTS was associated with a higher body weight and waist circumference^4^. Furthermore, higher CTS has been found to be associated with greater weight loss with the use of phentermine-topiramate in a 2-week randomized trial^4^. However, little is known about interindividual variability of human satiation and its long-term utility in the medical management of obesity.

Our study aims to explore the variability of human satiation and its potential implications for precision obesity management. Through this, we revealed that satiation is highly variable among individuals, and while baseline characteristics, anthropometrics, body composition and hormones, contribute to this variability, they alone cannot account for the totality of the variance in satiation. To address this gap, we explored the role of a polygenic risk score, which demonstrated a robust association with satiation. Furthermore, we developed a machine-learning gene risk score to predict satiation and to assess this score in the prediction of responses to anti-obesity medications.

## Results

### Understanding the Variability of Satiation

**Figure 1:**
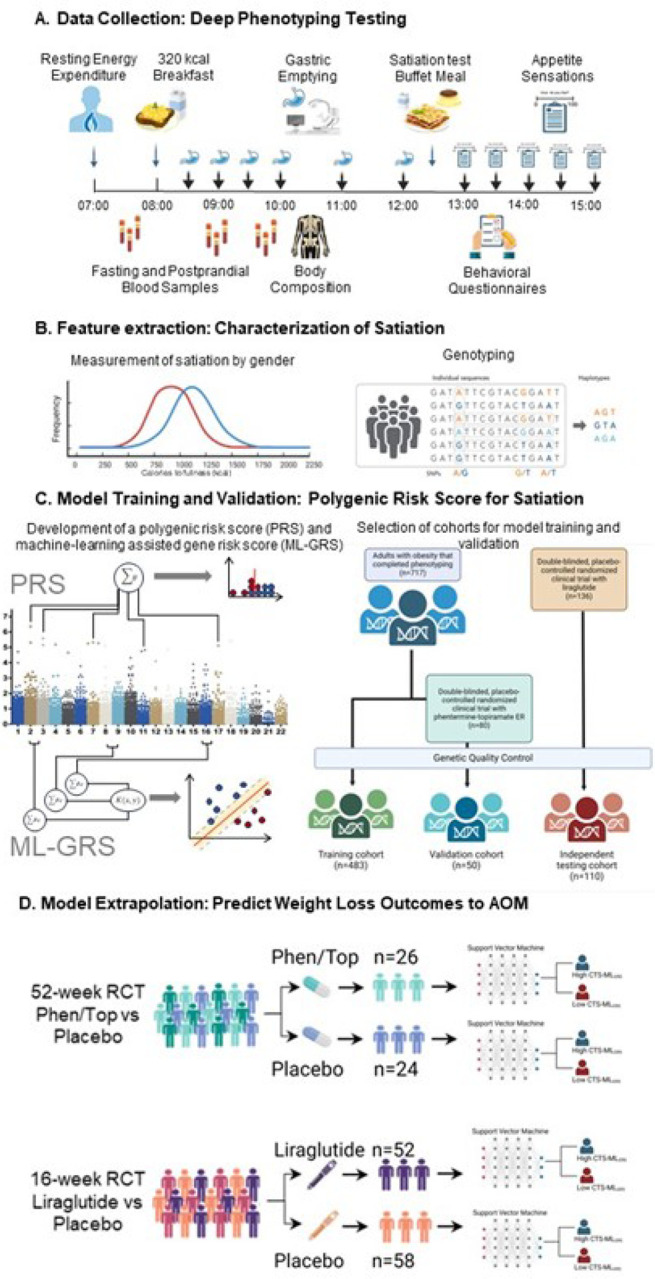
Experimental design. A) Data collection by deep-phenotype testing done in 717 participants. Illustrates the testing day starting at 7 a.m., including resting energy expenditure assessment, blood sampling, radio-labeled breakfast, gastric emptying scans, body composition by DEXA imaging, ad libitum meal test, and behavioral questionnaires. B) Feature extraction for satiation characterization. Shows male (n=179) and female (n=538) satiation distribution by calories to satiation, highlighting factors like hormones and genetics. C) Model training and Validation for Gene Risk Score Development. Demonstrates the creation and validation of a polygenic risk score (PRS) and machine-learning optimized gene risk score for satiation. D) Model extrapolation for Prediction of Response to anti-obesity medications. Depicts the model’s effectiveness in predicting response to obesity interventions using outcomes in a 52-week randomized, placebo-controlled trial of phentermine-topiramate ER (n=50) and a 16-week randomized, placebo-controlled trial of liraglutide (n=110) by categorizing participants according to CTS by ad libitum meal or by genotype groups.

In this study, our primary aim was to investigate the factors influencing the heterogeneity of satiation. We examined the baseline characteristics of adult participants with obesity (Body Mass Index [BMI] > 30 kg/m^2^) recruited between 2010 and 2021, all of whom underwent a comprehensive in-person deep-phenotyping with physiological and behavioral assessments to study components of energy balance regulation (Fig. 1A and methods). The deep-phenotype testing day started after an overnight fast and included the measurement of resting energy expenditure by indirect calorimetry, body composition by dual x-ray absorptiometry, the collection of blood samples, administration of a standardized radio-labeled 320-kcal breakfast to assess gastric emptying by scintigraphy and appetite sensations scales, measurement of satiation and behavioral questionnaires (Fig. 1A). In the satiation test, participants consumed an *ad libitum* meal consisting of vegetable lasagna, vanilla pudding, and skim milk. Nutritional analyses of the meal components were conducted, and participants were instructed to eat until they reached satiation, i.e., maximal fullness, assessed using a visual analog scale. The total kcal consumed to satiation (CTS) was the primary measurement. During the study day, blood samples were collected to extract DNA and plasma to measure fasting and postprandial gastrointestinal hormones (Fig. 1A).

**Figure 2:**
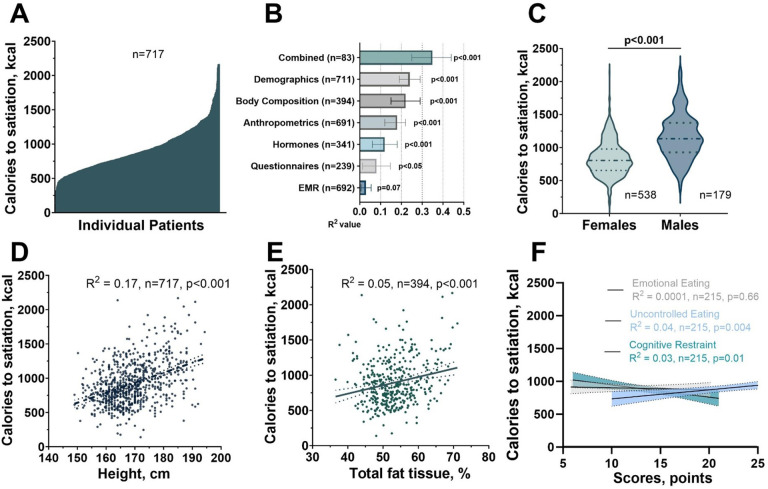
Comprehensive Analysis of Satiation Variability and Influencing Factors. A) Inter-Individual Variability in Satiation. This figure illustrates the distribution of satiation responses among 717 participants, highlighting the wide range of caloric intake to satiation. B) Proportional variance explained (R2) for satiation variability attributed to individual input variables, obtained from separate non-hierarchical regression models. Error bars represent the 95% confidence interval. R2 values were calculated through multivariable linear regression. C) Sex-Related Effects on Satiation Parameters. This violin plot showcases the distinct impact of sex on satiation parameters, revealing higher calories to satiation in males (p<0.001). D) Anthropometric Influence on Satiation. Univariate linear regression analyses display the weak correlation between calories to satiation and height in 717 participants. E) Body Composition Influence on Satiation. Univariate linear regression analyses display the weak correlation between calories to satiation and total fat percent in 395 participants that completed a DEXA scan. F) Questionnaire-Based Satiation Patterns. Demonstrates the limited contribution of behavioral questionnaires to explain the variability of objective measurements of satiation as demonstrated by the weak correlation between calories to satiation and scores in the components of the three-factor eating questionnaire: uncontrolled eating, cognitive restrain, and emotional eating completed in 215 participants.

The initial study cohort comprised a total of 717 participants that completed the full day of in-person deep-phenotyping. Participants were mostly females (75%), White (91.9%), had a mean age of 41.1 and standard deviation (±) 11.4 years and a mean BMI of 37.0 ± 7.1 kg/m^2^ (Supplementary Table 1). Females had a higher BMI in comparison to males (37.3 ± 7.0 kg/m^2^ vs 35.9 ± 7.4 kg/m^2^, p = 0.03). We observed satiation variability in our cohort, with a broad distribution among patients, ranging from 140 kcal to 2166 kcal to satiation (Fig. 2A).

To explore the factors contributing to the variability in satiation measurements, we conducted separate multivariable linear regression models for each group of variables. The groups encompassed demographic characteristics (i.e., age, sex, and race), anthropometrics (i.e., weight, height, waist circumference, and hip circumference), body composition (i.e., fat mass, lean mass, and fat free mass), hormones (i.e., peptide YY [PYY], ghrelin, and glucagon-like peptide 1 [GLP-1]), questionnaire data (i.e., Three-Factor Eating Questionnaire [TFEQ], Hospital Anxiety and Depression Scale, Alcohol Use Disorders Identification Test, and Weight Efficacy Lifestyle Questionnaire) and data on past medical history (i.e., past medical history of: diabetes, hypothyroidism, gastroesophageal reflux, metabolic dysfunction-associated steatosis liver disease, smoking, and history of use of anti-obesity medications). In addition, we conducted a single multivariable model that included demographic, anthropometric, body composition, and hormone variables (Supplementary Table 2).

Our analysis revealed that demographic characteristics, specifically sex, race, and age, explained the highest proportion of variance in satiation (r^2^ = 0.24; p = 0.001) (Supplementary Table 2; Fig. 2B). Despite these associations being statistically significant, the overall strength of the relationships remained weak across all models. Notably, a significant sex difference was observed, with females requiring fewer calories to reach satiation compared to males (835.2 ± 259.1 kcal vs. 1164.2 ± 340.5 kcal; p < 0.001) (Fig. 2C). Anthropometric variables explained 17% of the variance (r^2^ = 0.17, p = 0.001), with height explaining the most variance in satiation (r^2^ = 0.17; p = 0.001) within this category, although the amount of variability explained was small (Fig. 2D).

In the assessment of body composition, fat percentage demonstrated the strongest association with satiation among other parameters (see Supplementary Table 2). However, this association was still notably weak (r^2^ = 0.05; p < 0.001; Fig. 2E).

Despite our efforts to incorporate validated questionnaires, such as the TFEQ, into our analysis, it became evident that these components alone were insufficient to fully elucidate the variability in satiation (Fig. 2F). Similarly, a high degree of variability was observed in gastrointestinal hormonal Profiles, with limited associations with satiation, as demonstrated in Supplementary Table 3. Finally, a model incorporating demographic, anthropometric, body composition and hormone variables together had a modest association with satiation (r^2^ = 0.35; p < 0.001).

### Characterizing High and Low Calories Consumed to Satiation

**Figure 3:**
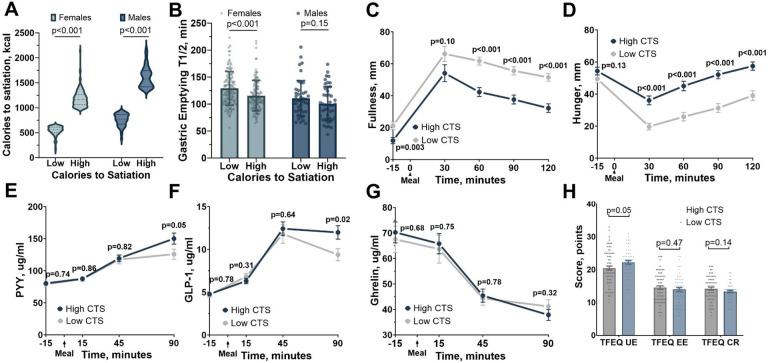
Multifaceted Insights into High and Low Calories to Satiation Phenotypes A) Comparative Analysis of High and Low Satiation Groups by Sex. This figure illustrates the comprehensive distinction between high and low calories to satiation, further categorized as high calories to satiation (n=179) with >977 kcal for females (n=134) and >1374 kcal for males (n=45), and low calories to satiation (n=161) with <650 kcal for females (n=134) and <927 kcal for males (n=44). B) Gastric Emptying Time by Gender in High and Low Calories to Satiation with significant differences in solid gastric emptying half-time in females (p<0.001) but not in males (p=0.15). C) Fullness by visual analog scale (VAS) after consuming a 320-kcal breakfast – High Calories to Satiation (n=70) vs. Low Calories to Satiation (n=79). D) Hunger Levels After a 320-kcal Breakfast - High Calories to Satiation (n=70) vs. Low Calories to Satiation (n=79). E) Profile of Gastrointestinal Hormones Fasting and Postprandial in High and Low Calories to Satiation: Profile of Peptide YY (PYY) Levels in High (n=83) and Low Satiation (n=59) showing not significant difference between groups at any time point. F) Profile of GLP-1 Levels in High (n=123) and Low Calories to Satiation (n=101) showing only significant differences between groups at 90 minutes. G) Profile of Ghrelin Levels in High (n=85) and Low Calories to Satiation (n=58) showing not significant difference between groups at any time point. H) Distribution of Eating Behavior Scores in the three-factor eating questionnaire (n=215) (cognitive restraint, uncontrolled eating, emotional eating) between High and Low Calories to Satiation.

Given the observed differences between sex (Fig. 2C), we stratified our participant cohort into quartiles based on their satiation responses, separately for males and females. Those falling within quartile 1, characterized by satiation responses of < 650 kcal for females and < 927 kcal for males, are hereafter referred to as low CTS. Conversely, participants in quartile 4, exhibiting satiation responses of > 977 kcal for females and > 1374 kcal for males, are referred to as high CTS (Supplementary Table 4 and Fig. 3A). Participants positioned in quartiles 2 and 3 were excluded from these analyses.

Females in the high CTS group had a faster solid gastric emptying half-time (115.4 ± 28.2 min) than the low CTS group (129 ± 31.2 min; p < 0.001); however, we did not observe a difference between high and low CTS in males (100.6 ± 31.6 min vs. 110.5 ± 32.9 min; p = 0.15) (Fig. 3B). After the standardized breakfast, participants with high CTS reported more hunger and lower fullness across all measured time points, except for fullness sensation at 30 minutes post-breakfast, when compared to participants with low CTS (Fig. 3C–D). In addition, participants with high CTS exhibited elevated levels of GLP-1 (11.98 ± 8.5 μg/ml vs. 9.36 ± 6.9 μg/ml, p = 0.02) and numerically higher PYY (150.10 ± 79.56 μg/ml vs. 125.82 ± 59.97 μg/ml, p = 0.05) 90 minutes after breakfast, with no significant changes in ghrelin secretion (Fig. 3E–G). There were no significant differences in validated questionnaires for TFEQ among groups (Fig. 3H). These findings emphasize the significant interpersonal variability of satiation and reveal that individuals with a high CTS are a distinct group with unique subjective and objective responses to the same meal, further emphasizing the complexity of satiation regulation.

### Association of Genetic variations and Satiation

Of the 717 participants, 483 had DNA available and passed the quality control for the genome-wide association study (GWAS). While acknowledging the discrepancy in scale compared to extensive cohorts in landmark obesity-related GWAS studies, which included up to 300,000 individuals^13–15^, our focus was on exploring potential genetic signals associated with CTS (Supplementary Fig. 1A). We did not observe a GWAS significant relationship between single nucleotide polymorphisms (SNPs) and CTS in participants with obesity. This limitation underscores the challenges of detecting subtle genetic signals in small cohorts and emphasizes the need for larger-scale investigations in the future. A volcano plot portraying the association between different genetic features and CTS among male and female subjects is presented in Supplementary Fig. 1B.

**Figure 4:**
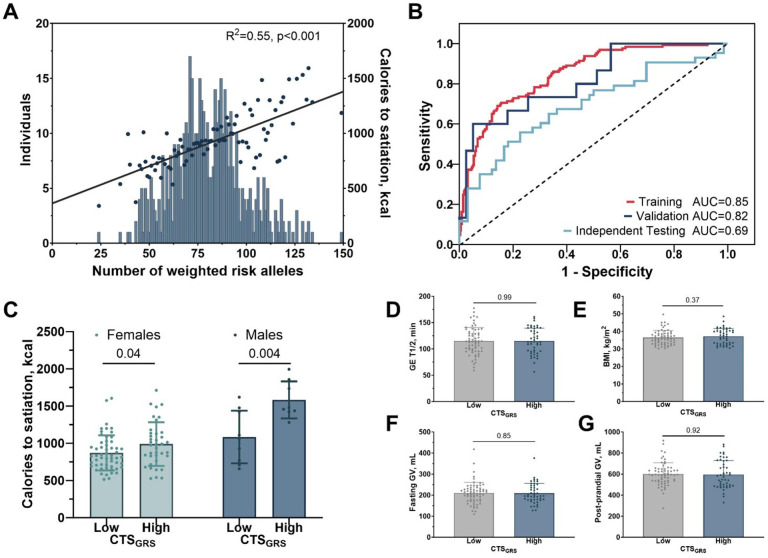
Development and Validation of a Machine Learning Assisted Gene Risk Score for High Calories to Satiation in People with Obesity A) Risk allele distribution for satiation. The number of risk alleles associated with higher calories to satiation was normally distributed in our population. Each circle represents the mean number of calories to satiation during the ad libitum meal test for each risk allele score. The solid line represents the linear regression for the risk allele score and calories to satiation (R2 = 0.55; β coefficient, 6.78; 95% CI, 5.46 to 8.11). B) Receiver Operating Characteristics curve for the Machine Learning Assisted Gene Risk Score in the training (n=483), validation (n=50), and independent validation cohort (n=110). C) Caloric intake in males and females according to their Machine Learning Assisted Gene Risk Score (CTS_GRS_) groups. Participants predicted as low CTS_GRS_ had a lower caloric intake than participants predicted as high CTS_GRS_. D-G) There were no differences in body mass index, or other parameters associated with postprandial satiety such as fasting gastric volume or post-prandial gastric volume when dividing participants according to their CTS_GRS_ group.

Consequently, we pursued a candidate gene approach^16^, focusing on genes associated with appetite regulation, energy expenditure, lipid metabolism, and adipogenesis identified in prior genetic studies. This targeted exploration yielded a curated set of 41 genes selected from a literature review and corresponding 19,161 unique annotated SNPs of which 2,637 were on the Omni Exome array (Supplementary Table 5). We selected 10% of the 2,637 SNPs that were the most correlated with CTS using Spearman correlation. This resulted in 227 candidate SNPs (Supplementary table 6). Subsequently, we developed a polygenic risk score (PRS) to predict CTS by summing the number of risk alleles across the 227 candidate SNPs. The PRS demonstrated a statistically significant correlation with CTS (R^2^ = 0.55; p < 0.001; Fig. 4A).

As a next step, we developed a machine-learning-assisted gene risk score (CTS_GRS_) to predict high CTS, where high denotes the quartile with the highest CTS (Q4) (Fig. 1C). We used machine learning algorithms to include other factors alongside genetics, which have been previously used to improve the predictive power of PRSs by accounting for non-linear genetic effects^17,18^. Specifically, we utilized demographic, anthropometric parameters, and genomic data from our participants with available genetic information (Supplementary Fig. 2). Our dataset was divided into training (483 participants) and validation (n = 50) cohorts (Supplementary Tables 7 and 8 and Supplementary Fig. 3). The validation cohort were those who participated in the 52-week, double-blind, placebo-controlled, randomized clinical trial comparing phentermine-topiramate extended-release versus placebo and completed phenotype testing (N = 62), and had available genetic data (n = 50) (Supplementary Tables 9 and 10 and Supplementary Fig. 4). Furthermore, an independent testing cohort, comprising participants from a 16-week, double-blind, liraglutide vs placebo-controlled, randomized clinical trial (n = 136) who had available genetic data (n = 110), was used for additional validation (Supplementary Tables 11 and 12 and Supplementary Fig. 5).

Random forests were used, and non-predictive features were removed by applying backward feature selection. We removed features until we reached a maximum training AUC, which was 0.81. This maximum performance used 16 GRSs representing 10 genes, including *SIM1, PCSK1, SH2B1, LEPR, UCP2, FTO, TCF7L2, GLP1R, TNFRSF11A*, and *ADRA2A* (Supplementary Fig. 6), and four demographic features, height, weight, sex, and age. We then evaluated these selected features using a Support Vector Classifier (SVC). An SVC using a linear kernel produced the highest training AUC and was thus used for the final model to predict CTS (CTS_GRS_) (Supplementary Fig. 2). This final model had a training area under the curve (AUC) of 0.85 (95% CI: 0.81 to 0.89), with a mean cross-validated AUC of 0.82 (95% CI: 0.67 to 0.95) across the 10-fold validation. In the validation cohort, the CTS_GRS_ achieved an AUC of 0.82 (95% CI: 0.69 to 0.94), while in the independent testing cohort, it attained an AUC of 0.69 (95% CI: 0.59 to 0.80) (Fig. 4B).

Participants in the independent testing cohort completed the deep phenotype testing day and additionally also completed testing for gastric volume and accommodation measured by noninvasive single photon emission computed tomography at baseline^4^. We tested the performance of the CTS_GRS_ in the independent testing cohort and found that individuals with a high CTS_GRS_ consumed more calories to satiation during the *ad libitum* meal in both females (988.9 ± 293.7 vs. 870.7 ± 236.0; p = 0.04) and males (1582.5 ± 239.9 vs. 1083.9 ± 353.8; p = 0.004) (Fig. 4C). It is worth noting that there were no statistically significant differences in BMI, gastric emptying for solids, and fasting and postprandial gastric volumes between high and low CTS_GRS_ prediction groups in the independent testing cohort (Fig. 4D–G). The results underscore the high specificity of our CTS_GRS_ model for satiation measurements, as there were no differences in anthropometrics, including BMI and other components that regulate food intake such as gastric volume, or emptying.

### Satiation Surrogate-biomarker and Weight Loss Interventions

To assess the clinical relevance of satiation, we investigated its role in response to two different weight loss interventions (Fig. 1D), phentermine-topiramate and liraglutide. Phentermine-topiramate extended-release (Qsymia^®^) was approved by the FDA for obesity in 2012. Phentermine suppresses appetite by releasing catecholamines in the hypothalamus, while topiramate enhances satiation and suppresses appetite through modulation of GABAergic pathways^19^. Phentermine-topiramate extended-release was classified as the most cost-effective medication to treat obesity^20^. Liraglutide (Saxenda^®^), FDA-approved for obesity in 2014, is a GLP-1 receptor agonists that induces postprandial satiety^19^.

First, we conducted a prospective assessment in a 52-week, double-blind, placebo-controlled, randomized clinical trial comparing phentermine-topiramate extended-release versus placebo. Treatment allocation was concealed, and investigators were blinded for the weight loss outcomes of patients with high or low CTS and CTS_GRS_. Then, we conducted a retrospective post-hoc analysis in a previously published 16-week, double-blind, placebo-controlled, randomized clinical trial comparing liraglutide versus placebo^6^. To study the effect of satiation and its surrogate genetic-based CTS_GRS_ biomarker, we included participants that completed the *ad libitum* meal, had available DNA data, and completed the trial. After stratifying participants by treatment allocation, we conducted independent t-tests at each timepoint to evaluate differences in total body weight loss across different groups.

**Figure 5:**
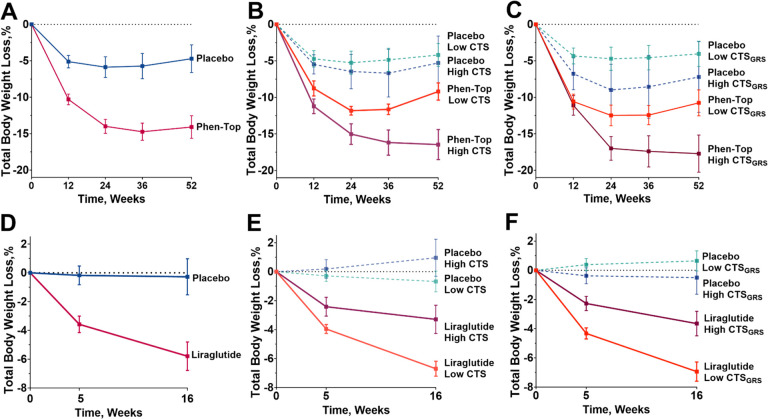
Weight loss response by baseline *ad libitum* meal energy intake and machine-learning assisted gene risk score prediction in response to placebo vs phentermine-topiramate ER and placebo vs liraglutide. A) Weight loss in all participants of a 52-week randomized clinical trial with placebo and phentermine-topiramate ER and underwent satiation testing at baseline. B) Weight loss by sex-stratified satiation by calories to satiation in the *ad libitum* meal in participants assigned to placebo and phentermine-topiramate ER, where participants assigned to phentermine and high calories to satiation by *ad libitum* meal had the greatest total body weight loss percentage. C) Weight loss by sex-stratified satiation by the machine-learning assisted gene risk score (CTS_GRS_) classified as low or high in participants assigned to placebo and phentermine-topiramate ER, where participants assigned to phentermine-topiramate ER and high CTS_GRS_ had the greatest total body weight lost percentage. D) Weight loss in all participants of a 16-week randomized clinical trial of liraglutide vs placebo and underwent satiation testing at baseline. E) Weight loss by sex-stratified satiation by *ad libitum* meal in participants assigned to liraglutide vs placebo, where participants assigned to liraglutide and low calories to satiation by *ad libitum* meal had the greatest total body weight loss percentage. F) Weight loss by sex-stratified satiation by machine-learning assisted gene risk score (CTS_GRS_) in participants assigned to liraglutide vs placebo, where participants assigned to liraglutide and low calories to satiation by CTS_GRS_ had the greatest total body weight lost percentage.

In the case of phentermine-topiramate versus placebo, a total of 62 participants that completed *ad libitum* meal with a mean age of 41.3 ± 9.8 years and a mean BMI of 38.6 ± 6.6 were included from the trial phentermine-topiramate versus placebo (Fig. 5A, and Supplementary Table 8). Individuals on phentermine-topiramate ER (n = 23) with high CTS during the *ad libitum* meal achieved better weight loss outcomes compared to those with low CTS (−15.6% ± 7.8 vs −9.2% ± 3.5; p = 0.03) at 52-weeks (Fig. 5B). There was no difference in weight loss outcomes at 52 weeks when comparing participants assigned to placebo with high or low CTS (p = 0.78). Next, we assessed the ability of predict the probability of achieving an equal to or greater than 15% total body weight loss. High CTS during the *ad libitum* meal predicted a probability of losing more than 15% of total weight at 52 weeks with an AUC of 0.82 (95% CI: 0.62 to 1.0; Supplementary Table 13A).

Similarly, in the cohort that underwent genetic testing, the CTS_GRS_ also proved useful in predicting the likelihood of successful weight loss in response to this intervention (Supplementary Table 9). At 52-weeks, participants on phentermine-topiramate ER with a high CTS_GRS_ lost more weight than participants with a low CTS_GRS_ (−17.7 ± 7.6 vs. −10.8% ± 5.6; p = 0.04), while all participants assigned to placebo experienced similar weight loss outcomes at 52-weeks regardless of the CTS_GRS_ (p = 0.49) (Fig. 5C). High CTS_GRS_ predicted a probability of losing more than 15% of total weight at 52 weeks with an AUC of 0.71 (95% CI: 0.42 to 0.99) (Supplementary Fig. 7).

In the trial of liraglutide versus placebo trial, a total of 136 participants with a mean age of 38.9 ± 10.6 years and a mean BMI of 36.5 ± 4.3 were included (Fig. 5D and Supplementary Table 10). Individuals with low CTS by the ad libitum meal achieved better weight loss outcomes compared to those with high CTS at 16 weeks (−6.7% ± 3.4 vs −3.3% ± 3.9; p = 0.005) (Fig. 5E). There was no difference in weight loss outcomes at 16 weeks when comparing participants assigned to placebo with high or low CTS. Low CTS by the ad libitum meal tests predicted a probability of losing more than 4% of total weight at 16 weeks with an AUC of 0.82 (95% CI: 0.62 to 1.0).

Similarly, in the cohort that underwent genetic testing (Supplementary Table 11), individuals with low CTS_GRS_ had superior weight loss outcomes in response to liraglutide at 16 weeks when compared to participants with a high CTS_GRS_ (−6.9% ± 3.4 vs. −3.7% ± 3.8; p = 0.004), whereas all participants assigned to placebo experienced similar weight loss outcomes at 16-weeks regardless of the CTS_GRS_ prediction (Fig. 5F). Low CTS_GRS_ tests predicted a probability of losing more than 4% of total weight at 16 weeks with an AUC of 0.75 (95% CI: 0.58 to 0.92) (Supplementary Table 13B and Supplementary Fig. 8). These findings underscore the potential of using satiation measurements from *ad libitum* meals or their corresponding genetic-based biomarker as unique predictors of the response to two specific anti-obesity medication with different mechanism of action.

## Discussion

This study conducted an in-depth characterization of human satiation in a cohort of 717 participants through physiologic and behavioral testing in a tightly controlled setting, revealing significant satiation heterogeneity. Our data highlighted that satiation, quantified as the calories consumed to reach fullness (i.e., CTS) during an *ad libitum* meal test, is influenced by a diversity of physiologic and behavioral factors. To further characterize satiation, we developed a polygenic risk score for CTS. We then employed machine-learning techniques to develop a machine-learning gene risk score for calories to satiation (CTS_GRS_). The clinical use of CTS and its surrogate CTS_GRS_ offer promising prospects for personalized pharmacologic interventions based on an individual’s satiation characteristics as supported by the data presented with phentermine-topiramate ER and liraglutide. Our findings support the clinical importance of tailored anti-obesity interventions using the key components of food intake regulation such as satiation.

The highly heterogeneous response to interventions in medicine has prompted the development of treatment strategies that are best suited to individual patients to enhance their effectiveness via precision medicine^21^. The variability in response to antiobesity interventions has been well documented in the literature over the past two decades^22^. Nevertheless, studies have underscored the challenges in identifying reliable predictors of weight loss response^23,24^. For instance, these studies have identified various predictors, such as short-term weight change, psychosocial factors, and health behavior change theories, but the accuracy of these predictions has remained limited. In contrast, our study offers promising prospects for precision pharmacologic interventions considering the heterogeneity of a key regulator of food intake: satiation. Importantly, we introduce the applicability of a surrogate CTS_GRS_ biomarker to predict satiation and thereby response to antiobesity medications.

Previous studies have used machine learning to predict food intake^25–27^. However, the particularity of our study lies in two facts. First, we focused on only one component of food intake regulation, satiation. Second, we jointly leveraged the current knowledge of the genetics of appetite regulation and our data regarding the association between calories to satiation and specific variants in order to develop a novel GRS. The relationship between genetics and satiation has been a subject of interest in the literature^28–30^. Studies exploring the complex interplay between genetic factors and satiation suggest that while genetic factors may play a role, environmental influences are also important contributors. The performance of our CTS_GRS_ is likely the reflection of this interplay and suggests that to improve its performance other environmental factors may need to be considered in future studies.

Our study extends beyond understanding the intricacies of satiation; it also explores the clinical relevance of satiation in the context of anti-obesity interventions. In a prospective assessment and in a retrospective post-hoc analysis of two randomized clinical trials, satiation measurement by calories to satiation based on the *ad libitum* meal and the surrogate CTS_GRS_ output predicted response to phentermine-topiramate and liraglutide. Interestingly, in individuals treated with phentermine-topiramate, higher CTS in the *ad libitum* meal and a high CTS_GRS_ both predicted greater weight loss compared to individuals with low CTS and low CTS_GRS_. Contrasting with liraglutide, that lower CTS in the *ad libitum* meal and a low CTS_GRS_ predicted greater weight loss in individuals treated with liraglutide compared to individuals with high calories to satiation. The differential response observed with these two medications deserves further studies to understand their unique mechanism of action within the selected pathways; nonetheless, a potential explanation may rely on the integrity of the GLP-1 receptor to melanocortin pathway to have an appropriate response to liraglutide, a GLP-1 receptor agonist. Supporting this hypothesis is the fact that higher number of risk alleles in this pathway correlates with higher calories to satiation; as well as previous studies showing that genetic variants in the GLP-1 receptor, only one gene in the pathway, influence liraglutide response^6,31,32^. Other key factors such as gastric function, vagal integrity, and reward pathways may be considered to explain these opposing results.

Predicting weight loss response refers to the ability to forecast how an individual will respond to a specific weight loss intervention, such as a diet, exercise program, or medical treatment^33–35^. Previous attempts at predicting weight loss response typically involved evaluating baseline characteristics, conducting health assessments, analyzing lifestyle and behavior, and considering psychological factors^36^. However, these efforts were often limited by the high volume of data input required and the low replicability they exhibited. Our study has elucidated a pathway that may predict the best responders to phentermine-topiramate or GLP-1 receptor agonist treatments through the utilization of a gene-based machine-learning algorithm. Although our current prediction capabilities remain confined to the cohort for which the model has been trained and tested, this represents a significant achievement. Furthermore, it paves the way for continuous learning and adaptation from long-term follow-ups, diverse cohorts, and, most importantly, learning from any encountered errors. As we expand our knowledge, we can refine and enhance our predictive abilities, ultimately offering more effective, personalized, and adaptable weight loss strategies.

This study has limitations. The use of the *ad libitum* meal in a clinical research unit setting raises concerns about the generalizability of satiation assessment. However, previous evidence suggests that this test is reproducible and has acceptable intra-individual variability^37^. Further, the CTS_GRS_ developed to predict satiation only incorporated genes that are linked to food intake regulation and obesity. As such, future larger studies should include GWAS to identify other potential genes linked to satiation and to validate the CTS_GRS_ in a more diverse population. Finally, the validation of satiation measurement by the *ad libitum* meal and the CTS_GRS_ which serves as biomarkers for satiation, as predictors of weight loss response to liraglutide involved a post-hoc analysis of a completed randomized clinical trial. While the independence of these cohorts has been strictly maintained for training the CTS_GRS_, further prospective randomized controlled clinical trials are crucial to validate the predictive utility of satiation measurement by *ad libitum* meal test and the satiation predicting CTS_GRS_.

In this comprehensive study, we highlight the heterogeneity of satiation. While gender-based differences, anthropometric measures, and hormonal factors played a role, they do not fully account for this heterogeneity. We demonstrate that genes involved in food intake regulation also partially account for this heterogeneity allowing the development of a CTS_GRS_ for predicting satiation. Furthermore, our study showcased the predictive utility of the CTS_GRS_ for individual responses to anti-obesity medications - phentermine-topiramate and liraglutide - underscoring its relevance for personalized obesity interventions.

## Methods

### Study population

Baseline characteristics were collected from participants with obesity recruited between 2010 and 2021 at Mayo Clinic, Rochester, MN. Eligible participants were adults aged 18 to 75 years, with obesity (BMI > 30 kg/m^2^), with or without type 2 diabetes, and otherwise healthy. Exclusion criteria included a history of abdominal surgery (except for appendectomy, laparoscopic cholecystectomy, cesarean section, or tubal ligation), chronic gastrointestinal diseases, systemic diseases affecting gastrointestinal motility, and the use of medications that may alter gastrointestinal motility, appetite, or absorption. Ethical approval for the use of data and samples from prior studies for the characterization and development of a biomarker was obtained from the Mayo Clinic Institutional Review Board (IRB#: 17–000838). All participants provided written informed consent for study participation, including the use of their genetic information in future studies.

#### Study Cohorts:

Our study included two distinct cohorts of participants. The initial study cohort comprised a total of 717 participants who completed a full day of in-person deep-phenotyping. This initial cohort was further divided into sub-cohort 1, utilized for training the machine-learning models, and a sub-cohort 2 used for validation of the models. The validation cohort consisted of 62 participants that also were part of the 52-week, double-blind, placebo-controlled, randomized clinical trial comparing phentermine-topiramate extended-release versus placebo (NCT04408586). Upon excluding related samples and samples from outlier genetic backgrounds, the validation cohort for the genetic models was refined to a total of 50 samples.

The second cohort consisted of 110 participants who underwent deep-phenotyping and were enrolled in a 16-week, double-blind, placebo-controlled, randomized clinical trial comparing liraglutide versus placebo^6^ (NCT03523273). This cohort served as the independent testing cohort.

Study Protocol for Deep Phenotyping and sample collection, and handling,

### In-person physiologic testing visit

Participants underwent screening to meet inclusion and exclusion criteria and were sent a pre-visit study pack containing questionnaires (Hospital Anxiety and Depression Scale [HADS] questionnaire, Questionnaire on Eating and Weight Patterns, and the Three-Factor Eating Questionnaire [TFEQ-R21]) to complete at home. Upon arriving at the Clinical Research Trials Unit (CRTU) at Mayo Clinic, Rochester, MN, between 6:00 a.m. and 8:00 a.m., participants were measured for heart rate and blood pressure while fasting. Standard clinical techniques were used to measure weight, height, hip, and waist circumference. Following the anthropometric measurements, participants underwent a baseline fasting blood draw, consumed a standardized radiolabeled breakfast to measure gastric emptying, and had an *ad libitum* meal for lunch (Figure 1A).

### Gastric emptying for solids

Participants received a standard breakfast comprising 320 kcal and 30% fat, including two 99mTc-radiolabeled eggs, toast, and 80 mL of skim milk. Images were taken immediately after ingestion and at regular intervals for a total of 4 hours. Gastric emptying (GE) was summarized by the half-emptying time (GE T1/2) in minutes^38^.

### Fasting and postprandial gastric volumes

Participants gastric volumes were measured by single photon emission computed tomography imaging (SPECT) of the stomach after intravenous injection of 99mTc-pertechnetate during fasting and after 300mL Ensure drink^39^.

### Satiation testwith *Ad libitum* meal

The *ad libitum* meal included vegetable lasagna [Stouffers^®^, Nestle USA, Inc, Solon, OH; nutritional analysis of each 326g box: 420kcal, 17g protein (16% of energy), 38g carbohydrate (37% of energy), and 22g fat (47% of energy)]; vanilla pudding [Hunts^®^, Kraft Foods North America, Tarrytown, NY; nutritional analysis of each 99g carton: 130kcal, 1g protein (3% of energy), 21g carbohydrate (65% of energy), and 4.5g fat (32% of energy)]; and skim milk [nutritional analysis of each 236mL carton: 90kcal, 8g protein (36% of energy), 13g carbohydrate (64% of energy), and 0g fat]. The total amount (g and kcal) consumed and the kcal of each macronutrient at the *ad libitum* meal were analyzed by a registered dietitian using validated software (ProNutra 3.0; Viocare Technologies Inc, Princeton, NJ). Participants were served the prepared meal. Subjects were asked to consume any food products until they achieved maximum satiation. A visual analog scale was used to assess maximal satiation (rate 0 to 5). As the subject completed each meal component, the empty plate, empty pudding container, and/or liquid volume of milk were weighed back. Once the subject reached maximum satiation, the total weight of food products consumed was summarized. The primary measurement was total kcal consumed to satiation (CTS).

### Eating behaviors

The Three-Factor Eating Questionnaire (TFEQ-R21) is a validated questionnaire designed to assess eating and weight control behaviors^40^. Participants completed the TFEQ-R21 on their baseline assessment visit. The TFEQ-R21 is a 21-item instrument that measures three domains of eating behavior: cognitive restraint (CR), uncontrolled eating (UE), and emotional eating (EE). The first twenty items are rated on a 4-point Likert scale, and item 21 is answered through an 8-point Likert scale. Before calculating domain scores, items 1–16 were reverse coded, and item 21 was recorded as follows: 1–2 scores as 1; 3–4 as 2; 5–6 as 3; 7–8 as 4. Domain scores were then calculated as a mean of all items within each domain; hence, domain scores ranged from 1 to 4 (CR [six items], UE [nine items], and EE [six items]), with higher scores being indicative of greater CR, UE, and EE.

### Blood Sample Collection

Informed consent was obtained from all participants. Baseline blood samples following an overnight fast were collected from each subject into 6ml EDTA blood collection tubes. The serum was kept at room temperature and immediately prepared for subsequent analysis of routine laboratory analyses or aliquoted and stored at − 80 °C until further analyses.

### DNA extraction

The samples were thawed at room temperature and a lysis buffer was added. The samples were then incubated at 56°C for 10 minutes. Proteinase K was added, and the samples were incubated at 56°C for an additional 30 minutes. Phenol/chloroform was added, and the samples were vortexed to mix. The samples were then centrifuged at 10,000 rpm for 10 minutes. The aqueous layer was transferred to a new tube and ethanol was added. The samples were vortexed to mix and then centrifuged at 10,000 rpm for 10 minutes. The supernatant was removed, and the pellet was air-dried. The pellet was then resuspended in the TE buffer. The DNA was purified using a commercial DNA purification kit. The kit included a lysis buffer, proteinase K, phenol/chloroform, and ethanol. The DNA was purified according to the manufacturer’s instructions. The DNA was quantified using a NanoDrop spectrophotometer. The DNA concentration was determined by measuring the absorbance of the DNA at 260 nm.

### Hormones

Levels of appetite-regulating hormones, Peptide YY (PYY), glucagon-like peptide-1 (GLP-1), and ghrelin, were measured in human blood samples using enzyme-linked immunosorbent assay (ELISA). Monoclonal antibodies specific to PYY, GLP-1, and ghrelin were immobilized onto microtiter plates, and the blood samples were added to allow hormone binding. Subsequently, a second enzyme-linked antibody was added, initiating a color-producing reaction proportional to the hormone concentration.

### Genome-Wide Association Study (GWAS)

Genotyping was carried out using the Illumina Infinium BeadChip technology. Quality control measures were implemented to ensure data accuracy and reliability, and samples with low call rates or ambiguous results were excluded from further analysis. Prior to analysis, further metrics were implemented to ensure only accurate genotype calls were used. We removed SNPs with a call rate < 0.95 and samples with call rate<0.95. The relatedness between each sample was assessed using the kinship coefficient. One subject from the following related pairs was retained: twins, parent/offspring, and full siblings. The sample with the higher call rate was retained. Genomic ancestry was assessed using STRUCTURE. Non-European samples were removed (e.g. >20% non-European). SNP flips between samples were handled automatically by the analysis software. We derived p-values for each SNP using a set of 483 samples. These samples have both genotyping data for 2.7M SNPs typed using Illumina’s Omni Exome array and CTS and exclude related samples and samples from non-European genetic ancestry (Supplementary Figure 2A). Using PLINK 2^11^, we performed linear regression using age as a covariate. Regression was performed separately for males and females as they have different CTS endpoints for high calories to satiation (Supplementary Figure 2B).

### Candidate Gene Selection

A set of candidate genes to build a polygenic risk score were selected from a literature review based on their biological relevance to obesity and previous evidence from genetic studies (Supplementary Table 5). Genes involved in appetite regulation, energy expenditure, lipid metabolism, and adipogenesis were prioritized for inclusion in the study. This resulted in a set of 41 genes and 19,161 unique annotated variants, of which 2,637 were SNPs contained in the Omni Exome array. Across all samples, we selected SNPs that were the most correlated with CTS (n = 227, r > 0.06) as those most likely involved in appetite regulation. Subsequently, a PRS for each individual was calculated by summing the number of risk alleles across the 227 SNPs.

#### Gene Risk Score (CTS_GRS_) Development

In preparation for training a machine-learning assisted gene risk score model to predict high calories to satiation (CTS_GRS_), we created a novel way of scoring of genes (Supplementary Figure 2). This score numerically represents the overall contribution of SNPs in and around a candidate gene to CTS and was done separately for males and females. To calculate this score, we investigated contributing factors at variants near the 41 candidate genes. These factors included the resulting beta value from linear regression analysis regressing SNP dosage on CTS (using sex-specific CTS endpoints) (Supplementary Figure 2B) and we weighted each SNP based on the proximity to the gene, and its presence within the gene (Supplementary Figure 7D). SNPs within one megabase pairs flanking each candidate gene were included (Supplementary Table 5). This process resulted in two numerical representations for each gene, which were separately derived for males and females based on the beta values from the endpoints of the GWAS. We refer to each numerical representation as a Gene Risk Score (GRS). There were two GRSs for each gene, one for males and one for females.

To develop a CTS_GRS_ to predict high CTS we considered the GRSs as well as demographic and anthropometric measurements (e.g., age, sex, weight). We used a random forest model with backward feature selection to identify the most informative features as measured by training AUC (see Supplementary Figure 2E). This method selected height, weight, age, sex, and 16 GRSs associated with 10 genes: SIM1*, PCSK1, SH2B1, LEPR, UCP2*, FTO*, TCF7L2*, GLP1R, TNFRSF11A, and ADRA2A. Genes marked with an asterisk had only the GRS derived from the female endpoint selected; see Supplementary Table 5 for the number of SNPs included in each GRS. Note that because of the algorithm’s greedy approach, features are selected when they are both predictive and contribute novel information. Thus, some expected genes, such as MC4R, were not selected because functionally related genes already contained similar predictive information, not because such genes were not predictive of CTS.

#### Machine-Learning Model Training to Predict High Calories to Satiation

The CTS_GRS_ was trained in 483 samples using a Support Vector Classifier (SVC) with the 16 selected GRSs, height, weight, age, and sex to predict high calories to satiation positive individuals (HS+). We defined HS+ as those individuals whose CTS was above the sex-specific 75th %ile (Male = 1372.4275 kCal, Female = 977.3325 kCal) and the others as HS-. Before training, we normalized the scores using a Yeo power transform^41^ and conducted SMOTE^42^ sampling to create 225 synthetic samples for training. We trained an SVC model 10-fold cross-validation on these oversampled data. For each fold, we t a Yeo power transform normalizer to the training data to normalize the fold’s train and test data (Supplementary Figure 2F). We measured a mean AUC = 0.82 across the 10 folds. Our final SVC model used all the training samples. All SVC models were trained using the Python sklearn.svm package with a linear kernel, C = 3, gamma = 0.01, and a balanced class weight. The final CTS_GRS_ model used oversampling and Yeo power transform normalization for all 483 training and 225 synthetic (708 total) samples and yielded both an HS+/− prediction and a probability of HS+ (p(HS+)).

#### Machine-Learning Model Validation

To evaluate our model, we tested the performance of our final SVC satiation model’s prediction accuracy on two placebo-controlled trials. These samples include 50 from a 52-week phentermine/topiramate trial (Test) which also underwent calories to satiation testing and had genotyping and demographic data. These samples’ data were normalized the Yeo power transformer fit to the 708 training samples and the HB+ class was set using the same 75^th^ %ile CTS thresholds. We then used the final CTS_GRS_ model to predict HS+/− using samples’ normalized data, evaluating the outcomes of the samples of the treatment arm of the study for those predicted to HS+ and HS− using a threshold of p(HS+) > 0.5 to define HS+. We measured the Total Body Weight Loss of individuals at 12-, 26-, 36-, and 52 weeks in each group under the hypothesis that predicted HS+ individuals are better responders (>15%TBWL at 52 weeks). Comparison of predicted and actual HS+/− shown in shown in Supplementary Table 7A.

#### Machine-Learning Model Independent Testing and Validation

We then measured the performance of our final SVC model’s predictions on the Liraglutide trial samples following the same procedures as the validation. These samples include 110 from a 16-week liraglutide trial (Validation) which also underwent calories to satiation testing and had genotyping and demographic data. To evaluate the outcomes of the samples of the treatment arm of the study for, we predicted to HS+ and HS− following the same procedure and threshold to define HS+ as with the Test set. We measured the Total Body Weight Loss of individuals at 5- and 16-weeks in each group under the hypothesis that, in this case, predicted HB− individuals are instead better responders (>4%TBWL at 16-weeks). Comparison of predicted and actual HS+/− shown in shown in Supp Tab 7B.

### Statistical Analysis

Continuous data were summarized as mean and standard deviation and categorical variables as percentages. Statistical testing included two-sample unpaired t-tests for Gaussian distribution and Mann-Whitney tests for non-Gaussian distribution. To explore the variability between satiation, as measured by the kilocalories consumed at an *ad libitum* meal to satiation, and a comprehensive set of exposure variables including demographics (such as sex, age, and race), body composition (comprising fat mass, lean mass, and fat-free mass), anthropometric measures (including weight, height, waist circumference, and hip circumference), hormones (including PPY, GLP-1, and ghrelin levels), electronic medical record-derived data (about medication usage and comorbidities), and responses to behavioral questionnaires, we employed a multivariable regression approach. The primary outcome of our analysis was the coefficient of determination (R2) to quantify the proportion of variability in satiation explained by these diverse factors. To assess the precision of our R2 estimates, we calculated the standard error using the formula SER2 = ((4R2 (1- R2)2 (n – k – 1)2) / ((n2 – 1) (3 + n)))1/2, where n represents the sample size and k represents the number of degrees of freedom in the model. Additionally, we reported 95% confidence intervals for R2, which were computed as R2 +/− 1.96 times the standard error.

After stratifying participants by treatment allocation, we conducted independent t-tests at each timepoint to evaluate differences in total body weight loss across different groups (high CTS vs. low CTS and high CTS_GRS_ vs. low CTS_GRS_). We did not use any method for data imputation for missing weight loss outcomes at different time points. The final model to predict CTS (CTS_GRS_), using all training samples, provided a continuous value ranging from 0 to 1, indicating the probability of having high CTS. Participants were dichotomously classified as having high if the probability was equal to or exceeded 0.5. We estimated the AUC by using the Wilson-Brown method for both CTS and CTS_GRS_ values in predicting the probability of achieving a weight loss equal to or greater than 4% and 15% of TBWL for liraglutide and phentermine/topiramate, respectively. To evaluate the real-world implications of the model’s predictions, t-tests were conducted to compare variables such as caloric intake, gastric emptying, and physiologic testing results among participants grouped by their predicted satiation groups.

We employed JMP Pro (Version 16.2.0., SAS Institute Inc, Cary, NC) and GraphPad Prism (Version 10.0, GraphPad Software, Boston, MA) for statistical analysis and visualization. Genome-wide association analysis was conducted using PLINK2. All models were developed and trained using Python.

## Data Availability

The raw data supporting the conclusions of this manuscript will be made available upon request to the corresponding author, without undue reservation, to any bona fide researcher with a methodologically sound proposal.

## Abbreviations

AUC: Area Under the Curve
BMI: Body Mass Index
CTS: Calories to Satiation
ER: Extended Release
GRS: Gene Risk Score
GWAS: Genome-Wide Association Study
GLP-1: Glucagon-Like Peptide-1
CTS_GRS_: Machine-Learning Assisted Gene Risk Score to predict high calories to satiation
PYY: Peptide YY
RCTs: Randomized Controlled Trials
ROC-AUC: Receiver Operating Characteristic Area Under the Curve
SNP: Single Nucleotide Polymorphism
TFEQ: Three-Factor Eating Questionnaire
VAS: Visual Analog Scales
